# Multicenter development and validation of a probability-based model to diagnose Lewy body disease using ¹²³I-*meta*-iodobenzylguanidine

**DOI:** 10.1007/s00259-025-07706-0

**Published:** 2026-01-14

**Authors:** Kenichi Nakajima, Junji Komatsu, Takeshi Matsumura, Satoshi Orimo, Mitsuhiro Yoshita, Viviana Frantellizzi, Maria Silvia De Feo, Gemma Greenfinch, Alan Thomas, Roberta Assante, Wanda Acampa, Naoki Shirasaki, Kunihiko Yokoyama, Hiroshi Wakabayashi, Moeko Noguchi-Shinohara, Kenjiro Ono, Seigo Kinuya

**Affiliations:** 1 Department of Nuclear Medicine, Kanazawa University Graduate School of Medical Sciences, 13-1 Takara-machi, 920-8640 Kanazawa, Japan; 2 Department of Neurology, Kanazawa University Graduate School of Medical Sciences, Kanazawa, Japan; 3 Kamiyoga-Setagaya Street Clinic, Tokyo, Japan; 4 National Hospital Organization (NHO) – Hokuriku National Hospital, Nanto, Japan; 5 Department of Radiological Sciences, Oncology and Anatomical Pathology, Sapienza University of Rome, Rome, Italy; 6 Institute of Nuclear Medicine, University College London Hospital, London, UK; 7 Translational and Clinical Research Institute, Faculty of Medical Sciences, Newcastle University, Newcastle upon Tyne, UK; 8 Department of Advanced Biomedical Science, University of Naples Federico II, Naples, Italy; 9 Department of Neurosurgery, Kaga Medical Center, Kaga, Japan; 10 PET Imaging Center, Public Central Hospital of Matto Ishikawa, Hakusan, Japan

**Keywords:** Sympathetic index, Parkinson disease, Dementia with Lewy-bodies, Diagnostic model

## Abstract

**Purpose:**

We previously proposed that a probability-based sympathetic ^123^I-*meta*-iodobenzylguanidine (*m*IBG) index (SMILe) could distinguish the presence or absence of Lewy body disease (LBD) based on findings at a single center. However, whether the model would be useful in the real world remained uncertain. Therefore, we updated and evaluated its performance at five Japanese and three European institutions.

**Methods:**

We compared data from 967 patients with suspected LBD with 62 controls from a normal database (NDB). Of 815 patients with guideline-based diagnoses, 483 had LBD (Parkinson disease [PD] or dementia with Lewy bodies [DLB]) and 332 did not have LBD. Heart-to-mediastinum (H/M) ratios were standardized using a phantom-based method. Logistic regression models included early and late H/M ratios, age, sex, and comorbidities. We assessed diagnostic performance using ROC analysis and cross-validation.

**Results:**

The updated model discriminated LBD from other diseases (AUC for early and late H/M, 0.880 0.899, respectively). Age correction of H/M ratios based on the NDB did not improve accuracy. Median early H/M ratios [SMILe probability] were 3.09 [12.8%] for NDB, 2.57 [37.5%] for Alzheimer disease, 1.76 [84.7%] for PD, and 1.62 [89.0%] for DLB, with significantly lower H/M ratios and higher probabilities in PD and DLB compared with controls (*p* < 0.0001). Late-phase imaging added value mainly in intermediate borderline (30%–70%) situations. Coronary artery disease attenuated the diagnostic performance of SMILe.

**Conclusion:**

The probability-based ^123^I-*m*IBG model reliably differentiated LBD from other diseases. Standardization among sites supports global applicability and reflects real-world clinical practice.

## Introduction

Since the 1990 s ^123^I-*meta*-iodobenzylguanidine (^123^I-*m*IBG) has been used to differentiate Lewy body diseases (LBD) such as Parkinson disease (PD), dementia with Lewy bodies (DLB), and pure autonomic failure from other neurodegenerative disorders presenting with symptoms of parkinsonism or dementia [1–3]. Since then, ^123^I-*m*IBG has become recognized as a biomarker reflecting cardiac sympathetic neurodegeneration associated with Lewy body pathology. Although its diagnostic utility has been established and neurological clinical guidelines have incorporated ^123^I-*m*IBG as a supportive tool for LBD diagnosis [4–6], universal consensus regarding optimal thresholds for distinguishing the presence and absence of LBD has not been reached. Reported cut-off values of the classical heart-to-mediastinum (H/M) ratio for diagnosing PD range from 1.34 to 2.4 for early and late phases with 82.6% sensitivity and 89.2% specificity [7]. A pooled meta-analysis of eight studies of < 80 patients each with DLB found high diagnostic accuracy, with 98% sensitivity and 94% specificity [2]. In contrast, the sensitivity and specificity of differentiating probable DLB from probable Alzheimer disease (AD) were 68.9% and 89.1%, respectively, according to a recent Japanese multicenter study [8]. These discrepancies highlight the key challenges conferred by the lack of standardization that results in a diagnostic “gray zone” where some patients cannot be classified with confidence. Furthermore, the feasibility and consistency of applying uniform diagnostic criteria in real-world clinical settings have not been fully evaluated, particularly when comparing clinical practices between Japanese and European institutions [9, 10].

We previously proposed that a sympathetic ^123^I-*m*IBG index for LBD (SMILe) with a probability-based approach could diagnose LBD and enhance the clinical applicability of ^123^I-*m*IBG cardiac imaging [11]. Unlike traditional dichotomous classifications based solely on H/M ratios, the SMILe index applies a logistic transformation to convert H/M ratios into the probability of LBD, which enables a clearer separation between patients with and without LBD. For example, if the threshold H/M ratio for discriminating the presence or absence of LBD was 2.2, H/M ratios of 2.3 and 2.1 would be respectively judged as negative and positive, while the probability values around the threshold would be intermediate. Our single-center study also found that early SMILe indexes could serve as useful adjuncts for diagnosis and identified a diagnostic uncertainty range of 0.3–0.7. Furthermore, late-phase images in this range improved diagnostic accuracy. Based on these findings, we revised the probability-based model and validated its ability to diagnose LBD. We leveraged a large database of patients from a total of eight Japanese and European institutions to determine the generalizability, diagnostic performance, and real-world applicability of this updated framework among diverse clinical settings.

The specific objectives of this study were to revise the SMILe index for probability-based LBD diagnoses, refine the prototype model, determine cross-regional generalizability, identify determinants of H/M variability (including clinical and technical factors), define whether the age-related decline of H/M ratio in the Japanese Society of Nuclear Medicine (JSNM) working group normal database (JSNM-NDB) [12, 13] warrants age adjustment, determine the incremental diagnostic value of late-phase images, and providing evidence-based thresholds for their use or safe omission.

## Methods

### Characteristics of updated database

This study analyzed data derived from a database created by a collaboration between five Japanese and three European hospitals. A total of 967 patients who were clinically suspected of having LBD were eligible for data analysis after applying the following exclusion criteria: familial amyloid polyneuropathy, outpatients referred from other hospitals solely for ¹²³I-*m*IBG examinations, and absent diagnostic followup. The reference controls were derived from the JSNM-NDB (*n* = 62). Thus, the final study population consisted of 1,029 individuals comprising 967 clinical patients with suspected LBD and 62 healthy controls from the JSNM-NDB (Table 1). This combination facilitated the development and validation of a probability-based diagnostic model in diverse cohorts from different sources.

**Table 1:** Patient demographics

	All clinical data	JSNM-WG NDB	Guideline-based data (Including NDB)
N	967	62	815
Age (y)	72.2 ± 11.1	57.0 ± 18.9	71.7 ± 12.2
Sex (male, %)	52.6%	45.2%	53.0%
Early H/M ratio standardized	2.22 ± 0.67	3.07 ± 0.50	2.19 ± 0.68
Late H/M ratio standardized	2.16 ± 0.86	3.25 ± 0.53	2.11 ± 0.84
Fraction of Lewy-body disease (%)	53.5%	0.0%	59.3%
Coronary artery disease*	2.9%	0.0%	2.5%
Heart failure*	0.8%	0.0%	0.1%
Diabetes mellitus*	8.4%	0.0%	7.3%

*H/M* heart-to-mediastinum ratio, *JSNM-WG* Japanese Society of Nuclear Medicine Working Group, *NDB* normal database, *Excluded Newcastle University data (*n* = 61)

The Ethics Committees at Kanazawa University and at the participating institutions approved this study. All data were transferred to a core laboratory (Kanazawa University) as a coded statistical data table render it innominate.

## Patient diagnosis

Among the 967 clinical patients, 815 were diagnosed according to internationally accepted clinical diagnostic guidelines for PD and DLB. Neurology specialists established diagnoses based on a comprehensive clinical evaluation that comprised detailed examinations of neurological symptoms and signs, as well as the integrated magnetic resonance, nuclear medicine, and x-ray computed tomography imaging findings that are required for final diagnoses. The diagnostic criteria for PD were clinically established probable PD based on the Movement Disorder Society criteria [4], and those for probable DLB were based on the Fourth Consensus Report of the DLB Consortium [5]. Nuclear medicine physicians at some institutions assembled databases by reviewing medical records, and diagnoses were based according to guidelines, or were tentatively described as clinical diagnoses in the absence of formal confirmation. Published data from a Newcastle University study were incorporated as part of the guideline-based dataset, and included information about patients with DLB and AD, as well as external controls. Notably, patients with diabetes and coronary artery disease (CAD) in the Newcastle dataset were not excluded, and six and one of them had a history of myocardial infarction (MI) and angina, respectively. However, because individual patient-level data were unavailable, these patients were included only to compare diagnostic accuracy and were excluded from analyses involving comorbidity.

The reference was data from the JSNM-NDB that was created based on information from nine hospitals [12, 13]. We rigorously selected patients to exclude those with known cardiac diseases, electrocardiographic (ECG) evidence of ischemia or infarction, wall motion abnormalities, arrhythmias inappropriate for ECG-gating, medications for hypertension or diabetes. The mean normal reference ranges of H/M ratios in early and late images were 3.1 (2.2–4.0) and 3.3 (2.2–4.4), respectively, under standardized conditions with a medium-energy general purpose (MEGP) collimator [14–16].

The final guideline-based diagnoses of LBD (*n* = 483) were PD (60%), DLB (39%), and rapid eye-movement (REM)-sleep disorders (1%). Patients without LBD (*n* = 332) had AD (36%), progressive supranuclear palsy (PSP) (11%), multiple system atrophy (3%), controls (including NDB, 28%), and other diseases such as mild cognitive impairment, vascular dementia, essential tremor, drug-induce parkinsonism, Huntington, and psychiatric diseases.

### Additional imaging data

We assessed dopamine transporter (DaT) abnormalities in a subset of 175 patients with 125 abnormal and 50 normal findings using ¹²³I-ioflupane imaging. These DaT imaging results were not used for primary classification but were incorporated as supportive diagnostic information in each participating hospital when establishing the final clinical diagnosis. Additionally, cerebral blood flow (CBF) SPECT images were acquired from 100 patients using N-isopropyl-(^123^I)-p-iodoamphetamine (^123^I-IMP), or ^99m^Tc-ethyl cysteinate dimer (^99m^Tc-ECD). We used the supplementary information to assist diagnostic decision-making. We did not directly compare analyses using either the DaT images or the CBF SPECT datasets.

### Comorbidities

We analyzed comorbidity data derived from the clinical records of 967 patients provided by the participating hospitals. Comorbidities were by comprehensive clinical evaluations and specialist judgment during routine workups. Patients from the Newcastle University dataset were excluded from this analysis due to the lack of individual data. The most prevalent comorbidities in the analyzed cohort were diabetes mellitus (DM), 8.4%, CAD, 2.9% and heart failure (HF), 0.8% (Table 1).

### ^123^I-*m*IBG imaging

All patients were evaluated by ¹²³I-*m*IBG cardiac imaging. We acquired images at 15–20 min (early) and at 3–4 h (late) after the Japanese and European patients were respectively injected with 111 MBq of MyoMIBG^™^ (PDRadiopharma Inc., Tokyo, Japan) and 185 MBq of AdreView^™^ (GE Healthcare, Chicago, IL, USA). Anterior images were acquired from supine patients at each institution using an Anger camera according to each protocol under the following parameters: matrix, 256 × 256; acquisition, 5–10 min; low-energy, low-to-medium-energy, or medium-energy general purpose (MEGP) collimators depending on institutional availability, and an energy window centered at 159 keV with a 15% window for all evaluations. Single-photon emission computed tomography (SPECT) images were also acquired at some institutions, but were not included in the primary analysis.

The H/M ratio was calculated by placing a circular or heart-shaped region of interest (ROI) over the left ventricle and a rectangular ROI over the upper mediastinum. We used smartMIBG software (PDRadiopharma, Inc.) for semi-automated ROI placement in Japan [17], whereas ROIs were manually selected and placed according to the proposed standardized method in Europe [18]. Because the participating institutions used different collimators, all H/M ratios were standardized to MEGP collimator conditions using phantom-based cross-calibration methods as described [14, 19]. This approach minimized camera and collimator-related variability and enabled direct comparisons among centers.

### Model development

A probability-based diagnostic model was developed as described [11]. Briefly, univariable and multivariable logistic regression were applied to the guideline-based dataset (*n* = 815) to estimate the odds of LBD. The logistic regression model was constructed as:

$$\:logit\left(P\right)={\beta\:}_0+\overset k{\underset{i=1}\sum}{\beta\:}_iX_i,$$

where $\:\mathrm{logit}\:\left(P\right)\:$= natural logarithm of the odds of having LBD (P/(1-P)), $\:{\beta\:}_{0}$ = intercept, $\:{\beta\:}_{i}$ = regression coefficients, and $\:{X}_{i}$ = predictor variables. The prototype SMILe model used coefficients of β_0_ = 5.932/4.232 and β_1_ = − 2.791/−2.184 for early/late imaging, respectively [11]. This model was originally derived from a cohort of 92 patients, comprising 29 with LBD (24 with PD and 5 with DLB) and 63 with non-LBD conditions. The predicted probability of LBD was calculated as:

$$\:\mathrm{P}=\:\frac{1}{1+exp(-\mathrm{logit}(P\left)\right)}\times\:100\%$$

This transformation converts the traditional H/M ratios and other predictors into a continuous probability score (%), which facilitated a more intuitive interpretation and provided the basis for the updated SMILe model.

### Data standardization and variables

The H/M ratios were standardized using collimator-specific correction factors derived from phantom calibration studies to account for inter-institutional variability among imaging protocols [14, 15, 20]. This standardization enabled direct comparisons of H/M ratios in the Japanese and European institutions. The probability-based diagnostic model was developed using multivariable logistic regression analysis and the predictor variables of early and H/M ratios, age, sex, and the comorbidities of CAD, DM, and HF.

### Impact of age and corrected H/M ratios

Myocardial ¹²³I-*m*IBG uptake gradually declines as age increases in the JSNM-NDB, and this is described as the following regression equations [13]:

$$\begin{array}{c}Early\mathit\;H\mathit/M\mathit\;ratio=3.39-0.00512\times Age\\Late\mathit\;H\mathit/M\mathit\;ratio=3.69-0.00711\times Age.\end{array}$$

We assessed the influence of age and age correction on the diagnostic performance of the model using an age-corrected H/M ratio that was normalized to the predicted value for a 60-year-old reference.

## Statistics

Continuous variables are presented as means ± standard deviation (SD) or as medians with interquartile ranges (IQRs), and distribution was visualized in box plots showing medians, 25th and 75th percentiles with whiskers representing minimum and maximum values. Mean values between groups were compared using one-way analysis of variance (ANOVA), followed by post-hoc pairwise comparisons using Tukey–Kramer honestly significant difference (HSD) tests. When distribution was skewed among the groups, differences among the five diagnostic groups were initially evaluated using Kruskal-Wallis tests. Significant differences were analyzed by post-hoc pairwise comparisons using Dunn tests for joint ranks. Holm-adjusted p-values were computed using Wolfram Language v. 14.3 (Wolfram Research Inc., Champaign, IL, USA) after exporting Dunn statistics to control for multiple tests. Categorical variables were analyzed using contingency tables with Pearson’s χ^2^ and likelihood ratio tests as appropriate. The diagnostic performance of the probability-based model was evaluated using ROC curves, and discriminatory power was assessed by calculating the area under the ROC curves (ROC-AUC). The optimal threshold for classification was determined using the point that maximized sensitivity – (1 – specificity) [21]. Univariable and multivariable logistic regression models were developed to predict LBD. All data were statistically analyzed using JMP software v.18.2 (SAS Institute Inc., Cary, NC, USA). Wolfram Language was applied to the logistic model and simulations that included probability curve visualization and logistic transformations. Values with two-tailed *P* < 0.05 were considered statistically significant.

## Results

### Distribution of H/M ratios

**Fig. 1:**
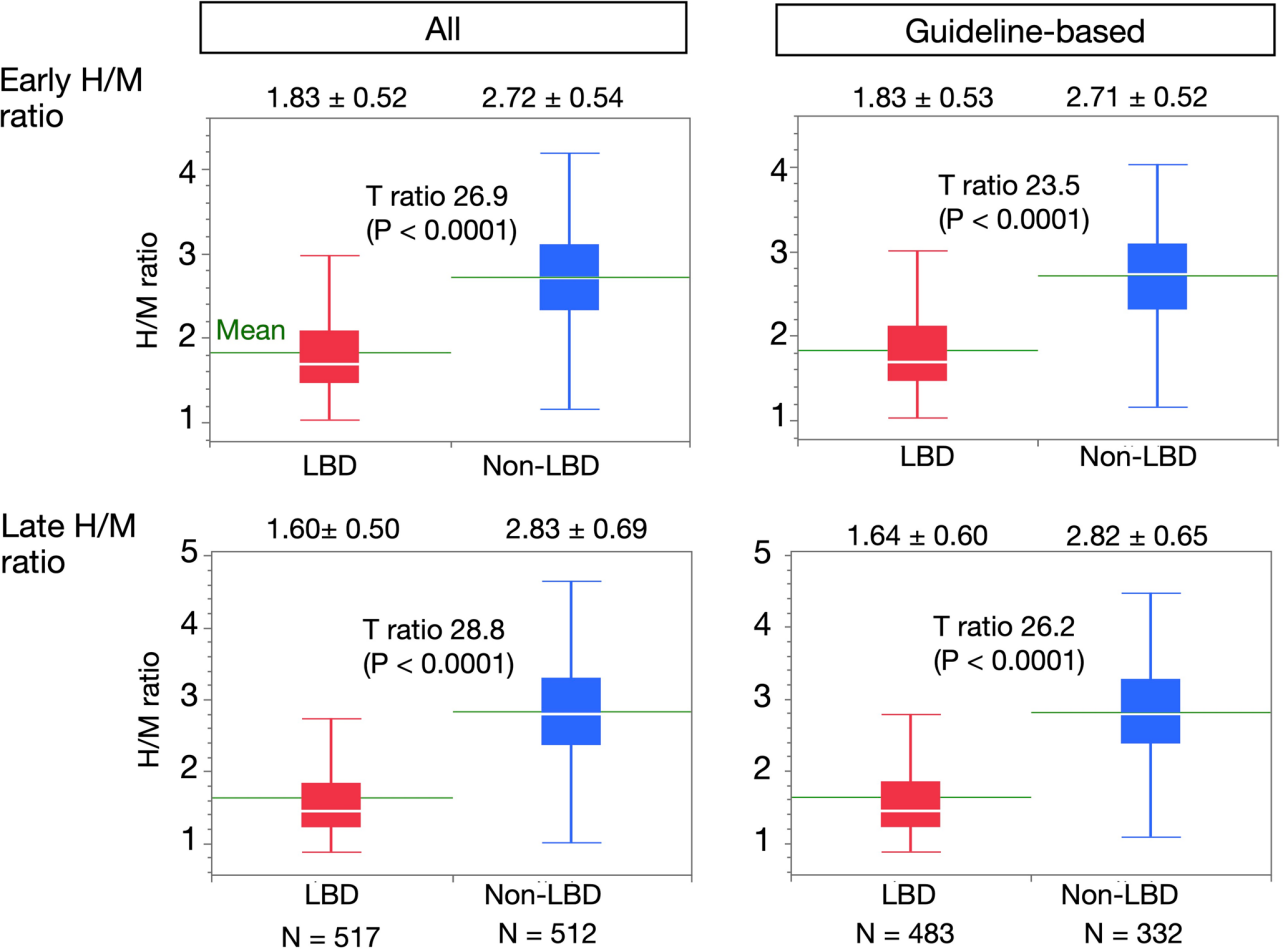
Early and late heart-to-mediastinum (H/M) ratios for patients with and without Lewy body disease (LBD). White (in red boxes) and green lines in box plots respectively indicate medians with interquartile ranges and means

We analyzed the distribution of early and late H/M ratios in patients with or without a diagnosis of LBD (Fig. 1). All enrolled patients (*n* = 1,029), including the JSNM-WG NDB, and the guideline-based diagnostic subgroup (*n* = 815), had been diagnosed according to the international criteria for PD and DLB. Early and late H/M ratios were significantly lower among patients with, than without LBD in both datasets (*p* < 0.0001 for both comparisons). These findings were consistent in the Japanese and European cohorts, indicating that the diagnostic separation based on H/M ratios is robust and globally reproducible.

### Comparison of Japanese and European groups

**Fig. 2:**
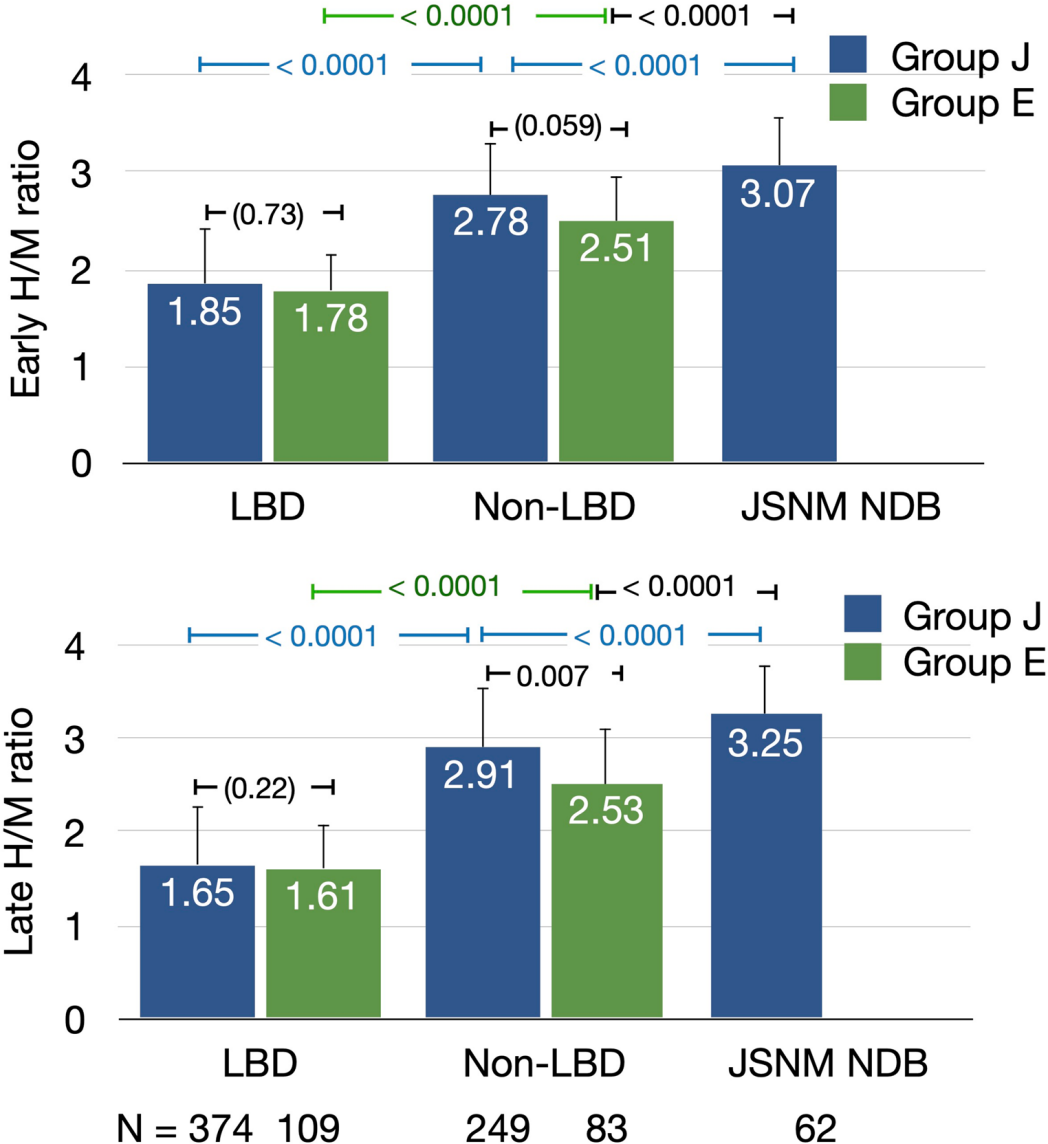
Comparison of early and late heart-to-mediastinum (H/M) ratios in patients with and without Lewy body disease (LBD). JSNM NDB, Japanese Society of Nuclear Medicine normal database

We compared the H/M ratios between Japanese (Group J) and European (Group E) patients with and without LBD (Fig. 2). Early and late H/M ratios did not significantly differ between the groups, indicating similar diagnostic performance among the regions. In contrast, late H/M ratios among patients without LBD were significantly higher for Group J than E on late images (*p* = 0.007), whereas the difference tended to be borderline for early images (*p* = 0.059, Tukey–Kramer HSD tests). Data from the JSNM-NDB revealed significantly higher H/M ratios than the Japanese and European patients without LBD (*p* < 0.0001), confirming clear separation of the normal reference values from persons with and without diseases.

### Logistic model

The univariable and multivariable logistic results showed that only early and late H/M ratios were significant for predicting a diagnosis of LBD (Table 2). The logistic regression model was applied to the guideline-based datasets to classify patients as having LBD or not. The key findings were the ROC-AUCs for early and late H/M ratios of 0.880 and 0.899, respectively. Incorporating age and presence of CAD into the multivariable model or age-corrected H/M ratios did not significantly improve the ROC-AUCs. These results indicated that early and late H/M ratios can differentiate patients with and without LBD, but the incremental value of explicit age correction was limited in this dataset. A comparison of diagnostic performance between the H/M ratio and the corresponding SMILe probabilities showed no change in ROC-AUC values, confirming equivalent discriminative ability.

**Table 2:** Univariable and multivariable logistic models using H/M ratios and other variables

	Estimate	SE	χ^2^	Odds ratio	Lower 95%	Upper 95%	*P*	ROC-AUC
**A. Univariable model with H/M ratios**								
**Early H/M**			386.2				< 0.0001	0.880
Early H/M	−2.737	0.183	222.5	0.065	0.045	0.092	< 0.0001	
(Intercept)	6.524	0.428	232.6				< 0.0001	
**Late H/M**			453.3				< 0.0001	0.899
Late H/M	−2.490	0.161	238.1	0.083	0.060	0.112	< 0.0001	
(Intercept)	5.814	0.372	244.8				< 0.0001	
**Early H/M-Age matched to 60 y**			384.4				< 0.0001	0.879
Early H/M	−2.744	0.184	222.0	0.064	0.044	0.091	< 0.0001	
(Intercept)	6.622	0.435	232.1				< 0.0001	
**B. Multivariable model**								
**Early H/M + age (Model summary)**			386.5				< 0.0001	0.880
Early H/M	−2.727	0.184	219	0.068	0.045	0.093	< 0.0001	
Age	0.0044	0.008	0.300	1.007	0.989	1.020	0.58	
(Intercept)	6.189	0.741	69.8				< 0.0001	
**Early H/M + age + CAD (Model summary)**			353.1				< 0.0001	0.882
Early H/M	−2.687	0.191	198.9	0.068	0.046	0.098	< 0.0001	
Age	0.0068	0.0081	0.710	1.007	0.991	1.023	0.40	
CAD	−0.052	0.294	0.030	0.901	0.266	2.742	0.86	
(Intercept)	6.081	0.815	55.6				< 0.0001	

*ROC-AUC* area under the curve of receiver-operating characteristic curve, *Η/Μ* heart-to-mediastinum, *SE* standard error

### Comparison of logistic curves: prototype vs. revised smile models

**Fig. 3:**
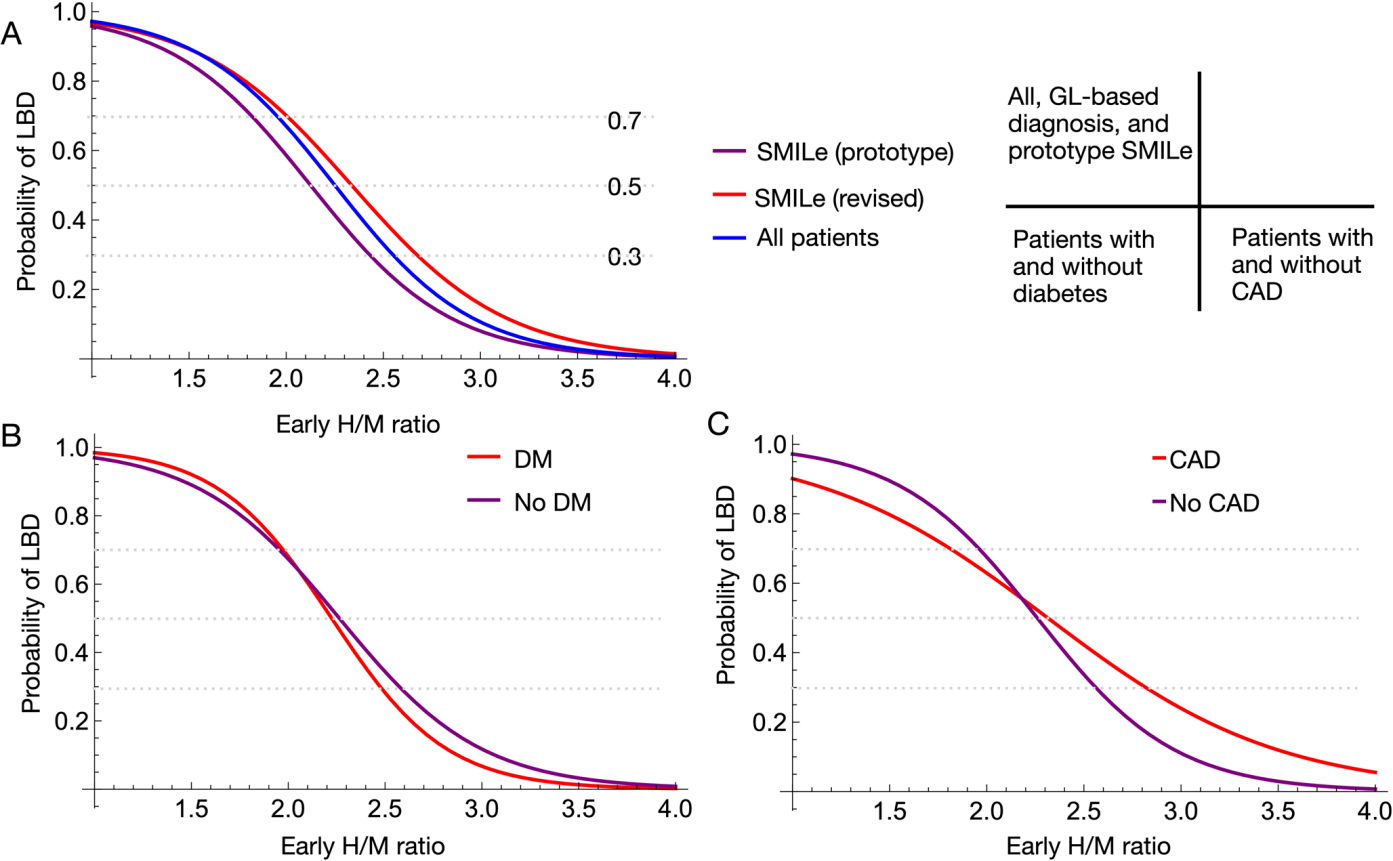
Logistic curve fitting for Lewy-body disease (LBD) probability versus early heart-to-mediastinum (H/M) ratios. (**A**) All patients, guideline (GL)-based database (revised SMILe) and prototype SMILe. (**B**) With and without diabetes mellitus (DM) and (**C**) with and without coronary artery disease (CAD). SMILe, sympathetic ^123^I-*meta*-iodobenzylguanidine (*m*IBG) index for Lewy body disease

We compared logistic regression curves derived from the published prototype (*n* = 92) and the SMILe (*n* = 815) models revised using the guideline-based dataset (Fig. 3). The coefficients used for the prototype and revised SMILe models were respectively − 2.791 and − 2.737 for β_1_, and 5.932 and 6.524 for β_0_. The logistic curve of the revised SMILe model shifted slightly rightward compared with the prototype, suggesting minor differences in the probability thresholds used to differentiate the presence or absence of LBD. Expanding the analysis to include all patients in the current (*n* = 967) and JSNM-WG normal (*n* = 62) databases led the logistic curve to lie between the prototype and revised models. This intermediate positioning reflected the greater variability introduced by including patients without guideline-based diagnoses, which slightly broadened the diagnostic probability distribution.

### Need for late imaging

**Fig. 4:**
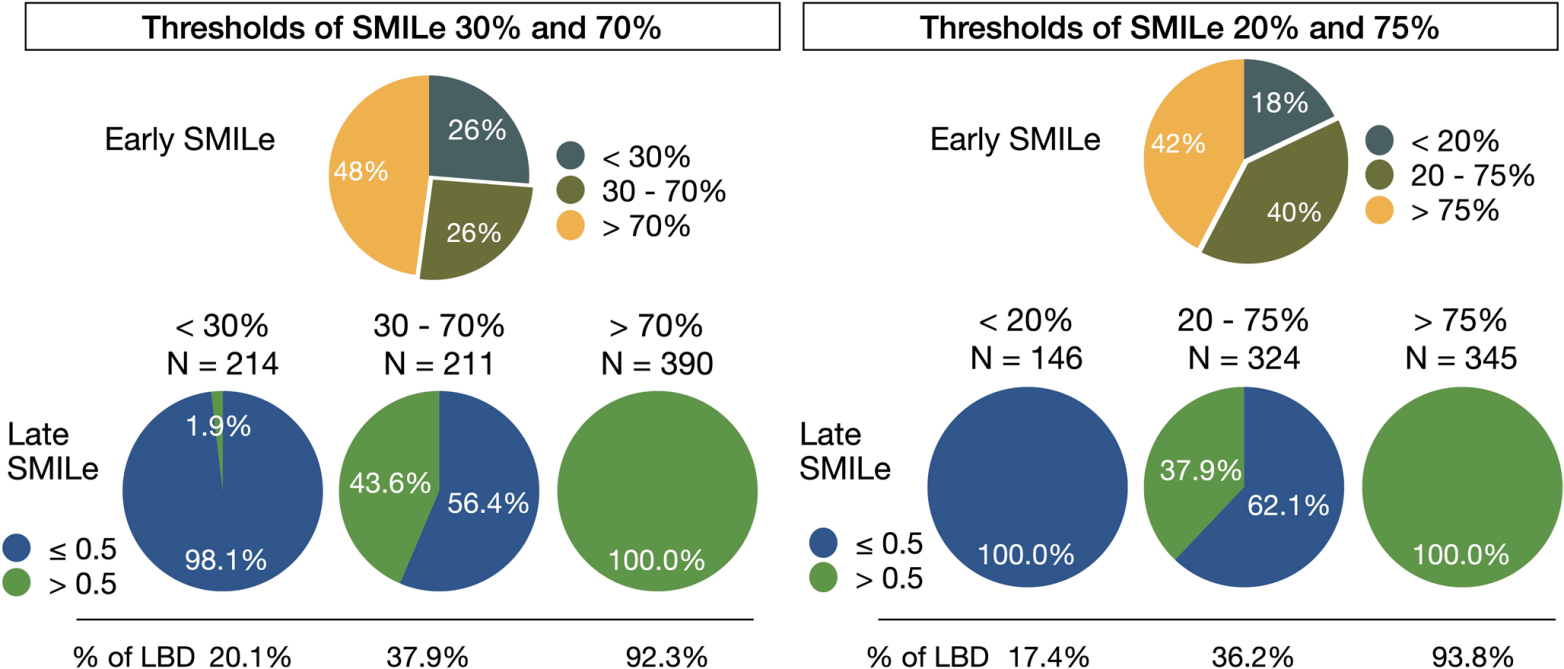
Fractions of patients using diagnostic thresholds of SMILe 30%/70% (left), and 20%/75% (right). Bottom panel shows fractions of late SMILe using 50% threshold. Fraction of final diagnosis of Lewy body disease (LBD) is shown in the bottom. SMILe, sympathetic ^123^I-*meta*-iodobenzylguanidine (*m*IBG) index for Lewy body disease

Patients were stratified according to early SMILe indexes of < 30%, 30%–70%, and > 70%. When the early SMILe indexes were < 30% and > 70%, the final diagnoses based on the late SMILe (< and ≥ 50%) changed in 1.9% (Fig. 4) and were not changed in all other patients, respectively. The final clinical diagnoses of LBD were confirmed in 20.1% and 92.3% of patients with early SMILe indexes of < 30% and > 70%, respectively.

Four patients had early SMILe indexes of 20%–30%, and late SMILe indexes that notably increased to 51%–63%, which led to a change in diagnosis (Table 3). These findings suggested that although late imaging rarely alters diagnostic outcomes when early SMILe is < 30% or > 70%, it is critical for patients within the intermediate probability range of 30%–70%, or are categorized as borderline probability. Adjusting the lower and upper SMILe thresholds to 20% and 75%, respectively, eliminated misclassification in this dataset.

**Table 3:** Four patients with early (< 30%) and late (> 50%) smile

Age (y) (sex)	CAD	HF	DM	Early H/M ratio	Late H/M ratio	Early SMILe	Late SMILe	Diagnosis early SMILe	Diagnosis late SMILe	Diagnosis Final
80 (F)	No	No	No	2.81	2.23	23%	53%	No LBD	LBD	AD
74 (M)	No	No	No	2.68	2.17	30%	56%	No LBD	LBD	PD
78 (M)	Yes	No	No	2.87	2.24	20%	63%	No LBD	LBD	PDD
65 (M)	No	No	No	2.80	2.26	24%	51%	No LBD	LBD	PD

*AD* Alzheimer disease, *CAD* coronary artery disease, *DM* diabetes mellitus, *F* female, *HF* heart failure, *H/M* heart to mediastinum, *LBD* Lewy-body disease, *M* male, *PD* Parkinson disease, *PDD* Parkinson disease with dementia, *SMILe* sympathetic *m*IBG index for LBD

#### H/M ratios and SMILe in major diagnostic groups

**Fig. 5:**
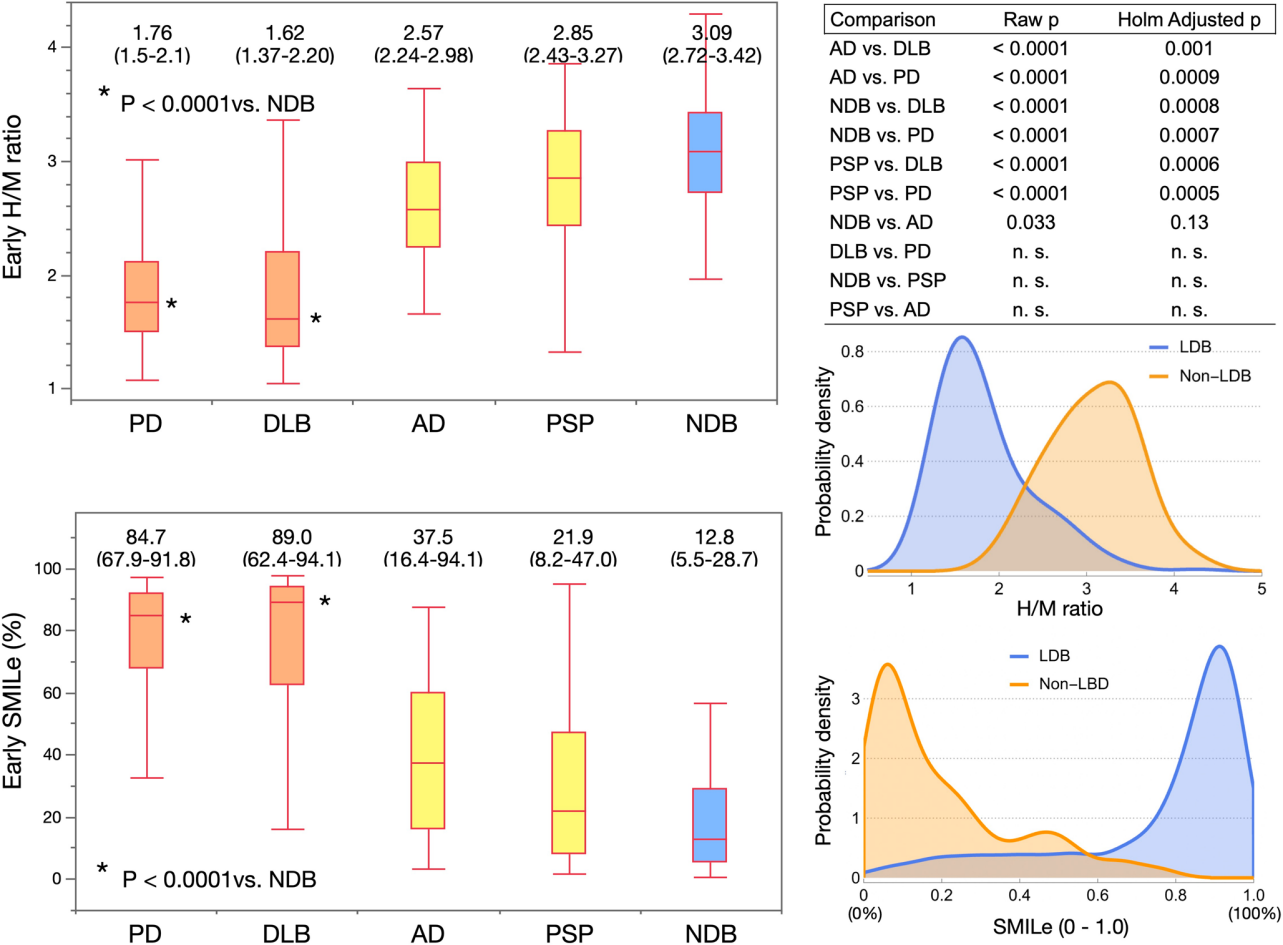
Early heart-to-mediastinum (H/M) ratios and SMILe probabilities for Parkinson’s disease (PD), dementia with Lewy bodies (DLB), Alzheimer’s disease (AD), progressive supranuclear palsy (PSP), and normal database (NDB). Right panel shows probability density functions for presence or absence of Lewy body disease (LBD) derived from H/M ratios and SMILe probabilities, with each curve normalized so that the total integral equals 1.0. SMILe distribution shows distinct peaks near 0.1 (10%) and 0.9 (90%), indicating clear separation between patients with and without LBD. Data are shown as medians and interquartile ranges (25%–75%). SMILe, sympathetic ^123^I-*meta*-iodobenzylguanidine (*m*IBG) index for Lewy body disease

The H/M ratios and SMILe values were analyzed in groups of at least 10 patients each from the JSNM-NDB (reference controls), and those with AD, PD, DLB, and PSP, based on early and late imaging results (Fig. 5). Median early SMILe probabilities for these groups were respectively, 12.8%, 37.5%, 84.7%, 89.0%, and 21.9%. Patients with PD and DLB showed significantly lower H/M ratios and higher SMILe values compared with NDB in both early and late images (adjusted *p* < 0.001 for all). In contrast, H/M ratios and SMILe indices did not significantly differ in patients with PSP compared with NDB. These results confirmed that the SMILe index effectively differentiates sympathetic-denervating disorders, such as PD and DLB, from controls and non-sympathetic neurodegenerative conditions.

## Discussion

This study is an extended validation of our proposed prototype SMILe model [11] from a single institution to a Japanese–European collaborative study involving eight institutions. We revised the probability-based diagnostic model using data from each institution and standardized ^123^I-*m*IBG measurements. The results showed that the revised model robustly differentiated patients with and without LBD among diverse populations. The revised SMILe model included a larger, guideline-defined cohort, which improved its stability and enhanced generalizability. Importantly, this is the first study to define the real-world international clinical use of ^123^I-*m*IBG cardiac imaging at eight institutions in Europe and Japan and show the feasibility of a probability-based diagnostic approach. We also revealed a diagnostic “gray zone” where late-phase images have meaningful incremental value, which supported a selective role in clinical applications.

### Advantage of probability-based diagnosis

Traditional cutoff-based diagnosis has been widely applied to ^123^I-*m*IBG imaging and other clinical applications, but such dichotomous thresholds have inherent limitations [10]. For example, the ADMIRE-HF trial proposed a threshold H/M ratio of 1.6 [22], which was subsequently adopted for patients with HF. However, Japanese and European multicenter studies then identified optimal thresholds of 1.68 and 1.75, respectively [23, 24], which emphasized inconsistencies among populations. Because prognosis and diagnosis cannot be reliably determined by a single cutoff value, reliance on fixed thresholds inevitably introduces ambiguity and potential misclassification. We have also proposed a multivariable (four/five-variable) probability model for predicting two- and five-year cardiac mortality in patients with chronic HF [25, 26]. This is similar in LBD diagnosis, in that thresholds from 1.6 to 2.2 [8, 27, 28] have been applied; however, borderline values remain difficult to classify.

In contrast, a probability-based approach provides a continuous diagnostic scale that helps clinicians to recognize when a patient lies in a borderline range, and to interpret such findings more intuitively with confidence. Furthermore, the probability model is flexible and expandable; additional variables can be readily incorporated into a multivariable framework to further improve diagnostic performance. Since the SMILe probability can be directly derived from the phantom-standardized H/M ratio [14, 19], it offers a practical tool for routinely estimating the likelihood of LBD in clinical settings. Importantly, this approach also resolves a long-standing practical issue by clarifying the circumstances under which late-phase imaging is necessary and justifiable [28, 29]. By clearly identifying when early-phase imaging is sufficient and when late-phase acquisition provides incremental value, physicians and technologists will be able to implement more personalized and efficient diagnostic strategies.

### Logistic model and optimal thresholds

**Fig. 6:**
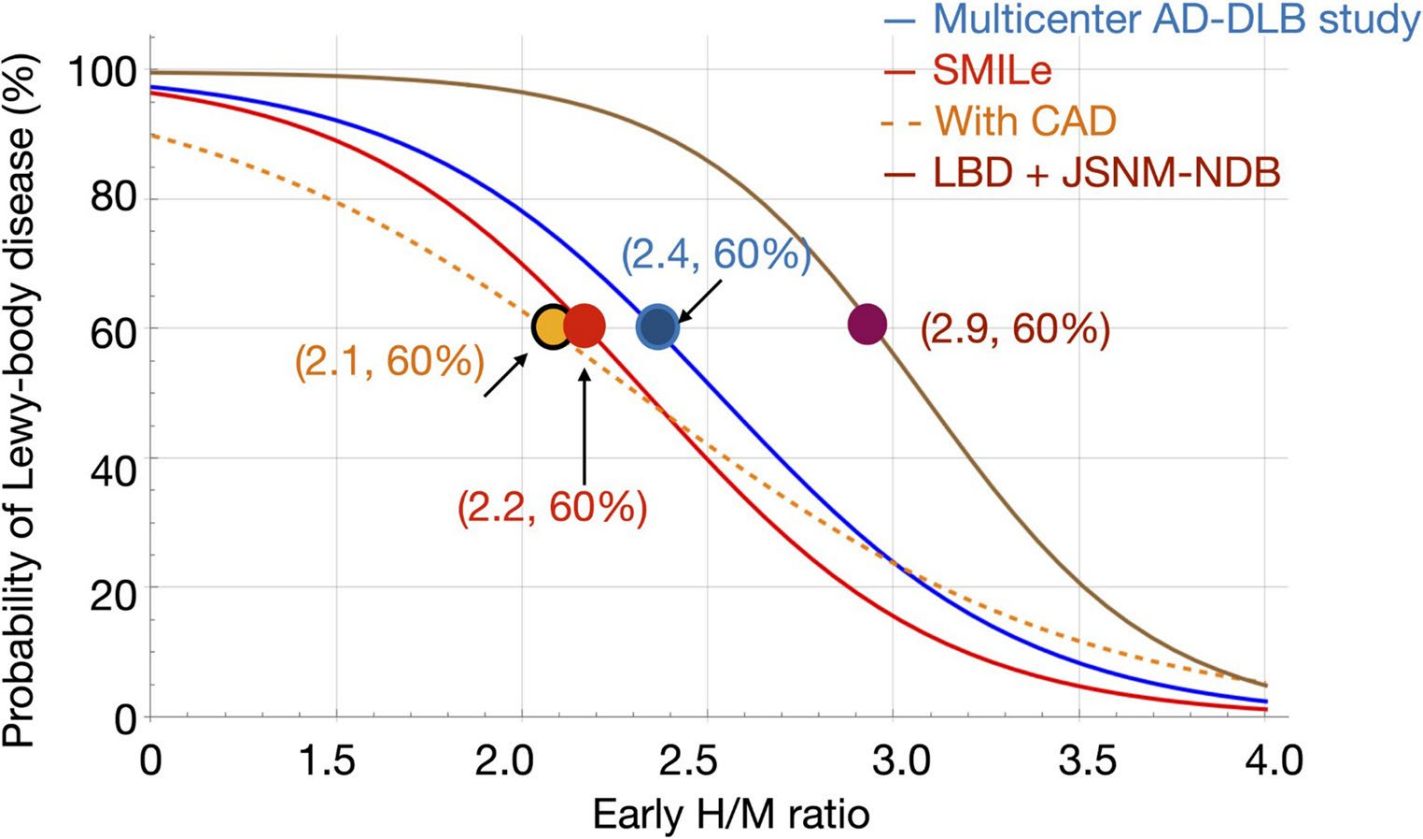
Comparison of logistic models under four clinical scenarios. SMILe model and cohorts without comorbidities (multicenter AD-DLB study [7]), with coronary artery disease (CAD), with Lewy body disease (LBD) plus Japanese Society of Nuclear Medicine normal database (JSNM-NDB). The presence of comorbidities such as CAD lowers the probability, while excluding comorbidities increases the estimated probability. As a result, the optimal cutoff point—for example, the threshold corresponding to an LBD probability of 60%—shifts depending on patient background, illustrating the advantage of understanding a probability-based diagnostic model over fixed heart to mediastinum (H/M) thresholds. AD, Alzheimer’s disease; DLB, dementia with Lewy bodies; SMILe, sympathetic ^123^I-*meta*-iodobenzylguanidine (*m*IBG) index for Lewy body disease

The variability in reported “optimal” thresholds among studies can be better understood by examining logistic models under different patient selection conditions (Fig. 6). When patients with CAD are included, the slope of the logistic curve flattens compared with the SMILe model, resulting in lower optimal thresholds. In contrast, when logistic models include only NDB and LBD patients, the curve shifts rightward and becomes steeper in the mid-range, yielding higher optimal thresholds. For example, in the Japanese multicenter DLB−AD study that excluded comorbidities, best thresholds were relatively high at 2.5 and 2.2 for early and late H/M ratios, respectively [8, 27]. Conversely, the optimal thresholds were lower (~ 1.8 and ~ 1.65) in the Newcastle study that included comorbidities [28]. These discrepancies reflect differences in patient inclusion criteria and the underlying probability distribution of LBD among cohorts. Figure 6 shows why cutoff values vary depending on patient background, referral and comorbidity profiles, and emphasize the strength of a probability-based diagnostic framework. By modeling continuous probabilities rather than relying on fixed thresholds, the SMILe model accommodates heterogeneity among populations and supports more robust, individualized diagnostic decision-making.

### Probability of LBD in major disease categories

This study also redefined the average SMILe probabilities across the major disease categories of AD, PD, DLB, PSP, and the JSNM-NDB. These updated probability estimates provide refined reference values that enhance the clinical interpretation of ^123^I-*m*IBG imaging results and facilitate more accurate comparisons of diseases. By establishing these standardized probability profiles, the present findings further support integration of probability-based diagnostic modeling into routine clinical workflows. This approach enhances diagnostic consistency and demonstrates applicability not only in research-focused analyses but also in real-world multicenter practice, where patient populations and imaging conditions are inherently diverse.

### Differences in ^123^I-*m*IBG index between Japan and Europe

We found slight, but statistically significant differences in H/M ratios among patients without LBD; H/M ratios were lower in Group E than Group J, whereas no significant difference was observed in patients with LBD in these groups. Several factors may explain these findings.

Technical factors: We standardized H/M ratios using phantom-based conversion to equivalent values generated using an MEGP collimator [14, 15]. This minimized instrumentation-related discrepancies in collimator conditions among institutions.

Patient comorbidities: Lower H/M ratios are prevalent in patients with DM, CAD, and HF, and are frequently associated with older populations. Although patients with severe DM and advanced HF were excluded, differences in local referral practices might have persisted. Physicians might have selectively excluded patients based on perceived indications for ^123^I-*m*IBG imaging. In contrast, patients with CAD were included, which could explain the slightly flatter logistic curve in the comparison of diagnostic models.

Body habitus: Height and weight data were not available in the current database. However, the prevalence of obesity is generally lower among Japanese patients. Greater body attenuation in some European patients might have potentially influenced image quality and measured H/M ratios.

Radiopharmaceutical factors: A contribution from ^123^I-*m*IBG specific activity cannot be excluded. Based on company-provided specifications, the specific activity of MyoMIBG^™^ ranges from 1,110 to 3,700 MBq/mL (https://pins.japic.or.jp/pdf/newPINS/ 00005871.pdf), whereas the corresponding value for AdreView^™^ is ~ 925 MBq/mL (https://www.accessdata.fda.gov/drugsatfda_docs/label/2020/022290s005lbl.pdf). The specific activity of ^123^I-*m*IBG is generally higher in Japan than in its European counterpart, although its impact on H/M ratios has not been formally verified.

These findings suggest that while technical harmonization was achieved, regional differences in patient characteristics, body habitus, and radiopharmaceutical properties might partially explain the modest discrepancies between Japanese and European patients without LBD.

### Indications for late-phase imaging

A wide variability in H/M ratios has been found between healthy persons and patients with LBD. Late-phase imaging has been routinely implemented in many institutions. However, our findings indicated that most patients fall outside the range where late images can influence diagnostic decisions. Omitting late-phase image acquisition is unlikely to compromise the diagnostic accuracy of ^123^I-*m*IBG imaging for patients with limited mobility or advanced dementia. Moreover, this approach can reduce the scan duration, minimize patient burden, and streamline workflow without sacrificing diagnostic reliability.

Using the SMILe probability model, we identified a practical diagnostic threshold between 0.3 and 0.7, within which, late-phase imaging provided the greatest benefit. More precisely, adjusting the probability range to 0.2–0.75 minimized the risk of misclassification to nearly zero, provided an evidence-based framework for optimizing imaging protocols and improving patient convenience.

The feasibility of omitting late images based on an H/M ratio threshold has been addressed in UK and Japanese studies [29, 30], but the diagnostic accuracy of the late H/M ratio was slightly better in a Japanese multicenter study [8, 27]. The results of the UK study that included patients with dementia and mild cognitive impairment indicated that when the early H/M falls between 1.70 and 2.50, patients can proceed to late imaging for confirmation; otherwise, early-phase imaging is sufficient and diagnostic classification is not changed. Our findings with the SMILe model are consistent with this diagnostic approach. Specifically, in our model, the UK thresholds of H/M = 1.70 and 2.50 corresponded to ~ 74% and 39% of LBD probability, respectively, within the probability-based framework (Fig. 6). For example, this finding notably highlights that an early H/M cutoff of 2.0 should not be interpreted as a definitive threshold, but rather as representing a patient-specific probability that depends on clinical context and study population characteristics.

The purpose of introducing these thresholds was not to apply a uniform rule to all patients, but rather to guide clinical decision-making, which is important. In the real world, ^123^I-*m*IBG imaging is often requested for atypical presentations to rule out LBD in patients with uncertain or mixed symptoms, and clinically suggestive findings, where imaging is used to confirm a strong suspicion of LBD. This streamlined approach might improve patient comfort and workflow efficiency, while preserving diagnostic reliability for patients in whom late imaging is most beneficial. Moreover, patients who fall into this gray-zone probability range might benefit from a more comprehensive clinical evaluation or, when appropriate, a follow-up ^123^I-*m*IBG study after ~ one year to confirm disease progression or stability.

### Influence of age on LBD diagnosis

Here, we did not identify age as a significant independent predictor for diagnosing LBD. Although we previously found a gradual age-related decline in H/M ratios using the JSNM-WG normal database, incorporating age into the prototype SMILe model did not improve diagnostic accuracy. This finding remained consistent in the present study, even after expanding the dataset from 92 patients in the prototype model to 815 in the guideline-based cohort. Therefore, age is apparently not a determinative factor for distinguishing patients with and without LBD.

### The H/M ratio in patients with AD

We found lower H/M ratios among patients with AD compared with controls, whereas others have found no significant differences between healthy persons and patients with AD. This discrepancy might be somewhat related to differences in baseline characteristics: the average controls in the JSNM-NDB were younger (mean age, 57 years) than the patients with AD (mean age, 75 years) and were specifically selected to exclude individuals requiring medications for cardiac or metabolic conditions. The group with AD included older patients with a higher likelihood of subtle comorbidities.

Moreover, incidental LBD is associated with various neurological conditions during the early disease process [31–33]. The frequent coexistence of Lewy body pathology with AD is now recognized as a mixed pathological condition. One possible explanation for the reduced ^123^I-*m*IBG activity in patients with AD is overlapping pathology, but this could not be fully assessed within the scope of the current database.

### Clinical implications for borderline findings

Our findings provide practical guidance for interpreting borderline results and informing clinical decision-making. In patients with very low (< 30%) or very high (>70%) early SMILe probabilities, late imaging provides minimal additional benefit, particularly for those with a high probability of LBD. The lower H/M ratios found in patients with AD or parkinsonism herein may reflect subclinical comorbidities or mild cardiac sympathetic dysfunction. Neurologists should carefully consider the potential influence of CAD and HF on borderline findings, as these conditions can lead to reduced late H/M ratios or an increased washout rate, even when early ^123^I-*m*IBG uptake is preserved. Indeed, low H/M ratios combined with high washout rates are established in patients with HF, particularly those at increased risk for lethal arrhythmic events [34, 35].

Taken together, these results support the applications and benefits of a standardized, probability-based diagnostic framework without requiring age correction. They also underscore the importance of integrating imaging findings with clinical assessments, particularly in diagnostically challenging patients where comorbid conditions may influence cardiac sympathetic imaging results.

### Limitations

This study has several limitations. The final diagnoses were determined within the context of routine clinical practice. A more robust diagnostic framework would ideally involve a centralized expert committee and systematic accumulation of comprehensive clinical information; however, establishing such a framework was beyond the scope of this study. Nevertheless, the dataset reflected real-world clinical workflows, which was the primary objective of this multicenter investigation. When very strict criteria are applied by excluding comorbidities, the logistic curve becomes steeper and shift rightward. Since the purpose of this study was to create a model for clinical practice, the present model may have practical significance. Supportive dopamine transporter and CBF SPECT images were available to only a few patients and we did not systematically analyze them. Comparative analyses integrating these modalities will be addressed in future investigations. Although our dataset included approximately 1,000 patients, larger patient cohorts will be necessary to enhance statistical power and validate the generalizability of the revised SMILe model. Moreover, the incremental clinical value of the SMILe probability framework compared with conventional H/M ratio thresholds awaits further evaluation in prospective studies. Although tomographic ^123^I*-m*IBG images may potentially enhance diagnostic accuracy, patients with typical LBD often show markedly reduced myocardial activity, which can make reliable reconstruction challenging. Tomography may instead be more useful for distinguishing LBD from comorbid conditions such as CAD or HF. Finally, exceptions can appear in clinical practice, particularly among patients with very rapid washout rates. Future studies incorporating washout kinetics might help to refine probability-based diagnostic thresholds in such scenarios.

## Conclusion

We updated the SMILe model to enable a probability-based approach to diagnosing LBD using ^123^I-*m*IBG cardiac imaging. By expressing diagnostic likelihood as a continuous probability, this model provides a clearer and more intuitive understanding of disease probability and simplifies differentiating the presence or absence of LBD. The revised model also established practical guidance for the selective use of late-phase images, and identified patients who would benefit the most as well as those for whom it can be safely omitted. Our findings support the integration of the SMILe probability framework into real-world clinical workflows, while emphasizing the need for further investigation to validate its broader applicability and determine additional clinical utility.

## Data Availability

The datasets generated and/or analyzed in the current study are not publicly available due to the approval of Ethics Committee but are available from the corresponding author on reasonable request.

## Abbreviations

AD: Alzheimer disease
CAD: Coronary artery disease
CBF: Cerebral blood flow
DLB: Dementia with Lewy bodies
DM: Diabetes mellitus
HF: Heart failure
H/M: Heart-to-mediastinum
JSNM: Japanese Society of Nuclear Medicine
LBD: Lewy body disease
*m*IBG: *Meta*-iodobenzylguanidine
NDB: Normal database
PD: Parkinson disease
PSP: Progressive supranuclear palsy
SMILe: Sympathetic mIBG index for Lewy body disease
